# Eco‐Friendly Biomass‐Based Carbon Dots, Carbon Nanotubes, Graphene, and Their Derivatives for Enhanced Oil Recovery: A New Horizon for Petroleum Industry

**DOI:** 10.1002/open.202400353

**Published:** 2025-04-30

**Authors:** Ruhul Amin Foisal, Abu Bin Imran, Al‐Nakib Chowdhury

**Affiliations:** ^1^ Department of Chemistry Bangladesh University of Engineering and Technology Dhaka 1000 Bangladesh; ^2^ Department of chemistry Bangladesh University of Engineering and Technology Dhaka 100 Bangladesh; ^3^ Department of chemistry Bangladesh University of Engineering and Technology Dhaka 100 Bangladesh

**Keywords:** Green Enhanced Oil Recovery (GEOR), Enhanced Oil Recovery (EOR), Carbon Dots (CDs), Carbon Nanotubes (CNTs), Graphene, Nanomaterials, Oil Recovery Efficiency, Nanotechnology

## Abstract

Oil extraction from reservoirs has never been easy, particularly when easily accessible oil sources run out. Enhanced oil recovery (EOR) is a dynamic area of petroleum engineering that seeks to maximize the quantity of crude oil that can be retrieved from an oil field. Researchers and oil producers have emphasized assessing tertiary‐stage recovery approaches, such as chemical EOR (CEOR), due to the problems posed by the diverse carbonate rocks. Polymers and surfactants used in CEOR procedures have the potential to harm formation and contaminate the environment. The environmentally beneficial “green enhanced oil recovery” (GEOR) technique includes infusing green fluids to raise tertiary oil output and boost macroscopic and microscopic sweep efficiency, ensuring sustainable practices while minimizing environmental concerns. Utilizing eco‐friendly carbon nanomaterials such as biomass‐based carbon dots (CDs), carbon nanotubes (CNTs), graphene, and their derivatives for EOR and reservoir monitoring applications represents a promising frontier in the petroleum industry. These particles are pricey and do not extend to GEOR but have been successfully tested in EOR. This innovative approach capitalizes on the unique properties of these nanomaterials to improve the efficiency and sustainability of oil extraction processes. This review aims to explore biomass‐derived carbon nanoparticles and investigate their possible functions in GEOR. Furthermore, the use of carbon particles in the GEOR approach is still poorly understood; thus, there needs to be a lot of credentials. The effectiveness, sustainability, and environmental responsibility of petroleum production operations can be enhanced by incorporating carbon nanomaterials from biomass into enhanced oil recovery systems. An environmentally friendly and more resilient energy future may be possible if research and development in this area are allowed to continue. This might completely change how oil resources are found and used.

## Graphical abstract

This review explores the integration of biomass‐derived carbon nanomaterials in green enhanced oil recovery (GEOR) techniques. It highlights the challenges of conventional chemical EOR, emphasizing the environmental benefits of carbon dots, carbon nanotubes, and graphene derivatives. The study investigates the potential of these nanomaterials to improve oil recovery efficiency while promoting sustainability in petroleum extraction practices.

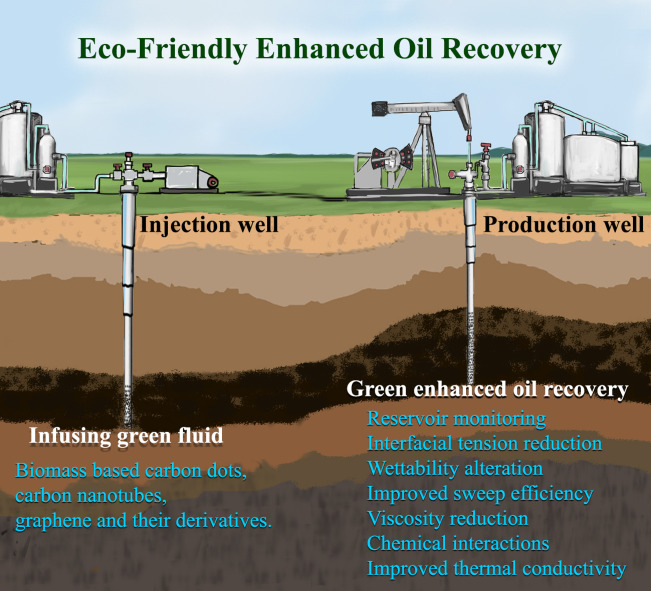

## 1 Introduction

Around the globe, primary and secondary recovery techniques are estimated to have retrieved around one‐third of the original oil in place (OOIP), leaving reservoirs with between 60 % and 70 % of the oil still in them.^[1]^ Most of the oil produced today originates from developed fields with significant residual oil content. The main goal for petroleum companies and governments worldwide is to increase oil recovery from these mature fields. Enhanced oil recovery (EOR) aims to maximize oil extraction from existing reservoirs. Traditional EOR methods include thermal recovery, gas injection, and chemical flooding. Recently, advanced carbon nanomaterials, including carbon dots (CDs), graphene, carbon nanotubes (CNTs), and their derivatives, has the potential for the EOR technique due to their unique characteristics. These carbon‐based nanomaterials are increasingly being explored for EOR to maximize crude oil extraction from reservoirs.[^2^, ^3^] Biomass‐derived carbon nanomaterials offer green, cost‐effective, and sustainable alternatives with superior properties like large surface area, low toxicity, and good biocompatibility, making them promising for GEOR in challenging reservoirs, especially for heavy oil resources.[^3^, ^4^] The application of nanofluids and nanocomposites, which often include carbon‐based nanoparticles, has potential to increase oil recovery rates by up to 79 %, depending on the specific nanomaterial used.^[5]^ These materials, synthesized from plant biomass using various methods like pyrolysis and ball‐milling, improve oil recovery efficiency through smart water and green chemical flooding techniques.^[6]^ For example, using nanofluids and nanocomposites containing graphene oxide and silica nanoparticles has significantly enhanced reservoir wettability, improving oil recovery rates under harsh conditions.^[7]^ Furthermore, nanomaterials can enhance recovery mechanisms by altering rock wettability, reducing interfacial tension, increasing fluid viscosity, and promoting spontaneous imbibition, ultimately improving sweep efficiency and overall recovery rates.^[2]^ However, challenges such as limited field data and the need for further research to optimize nanomaterial performance in unconventional reservoirs remain, highlighting the importance of ongoing studies to unlock the full potential of these advanced carbon nanomaterials in EOR applications .[^2^, ^3^] By leveraging these innovative materials, the petroleum industry can explore more efficient and environmentally friendly approaches to maximize oil production while minimizing environmental impact.^[8]^ This article discusses the use of advanced carbon nanomaterials in GEOR and offers a comprehensive overview of their synthesis, mechanisms, benefits, challenges, and future directions.

## 2 CDs and their Derivatives in GEOR

CDs, nanoscale carbon‐based materials with exceptional photoluminescent properties and surface functionality, can be derived from various biomass sources, offering environmentally friendly alternatives to traditional carbon sources.^[9]^ Biomass‐based CDs have shown significant potential in reservoir oil recovery applications. These CDs, derived from guava and date leaves, enhance oil recovery processes. They have been utilized in smart water flooding and green chemical flooding methods, showcasing increased oil recovery efficiency compared to traditional methods.[^6^, ^10^] CDs′ small size (2‐10 nm) and high surface area‐to‐volume ratio make them ideal candidates for applications in EOR, where they can serve as stabilizers or surfactants in recovery fluids.^[11]^ Their superior optical properties, biocompatibility, and cell penetrability further enhance their potential in this field, enabling efficient interaction with oil molecules to improve the displacement and recovery of trapped oil in reservoirs.^[11]^ Additionally, tests conducted on cores show that added CDs may be almost entirely recovered without harming carbon capture and storage (CCS) reserves.^[12]^ CDs can track the routes of CO_2_ injection, and coating their surfaces might improve their functionality in carbonate deposits.^[12]^ CDs′ sustainable nature, non‐toxicity, and biocompatibility make them promising tools for diverse applications, including sensing, biology, and bioimaging.^[9]^ These potential applications demonstrate that GEOR′s research on ecologically friendly biomass‐based CDs has become a novel and fascinating area in materials science and sustainable energy.

### 2.1 Synthesis of Eco‐Friendly Biomass‐Based CDs

**Figure 1:**
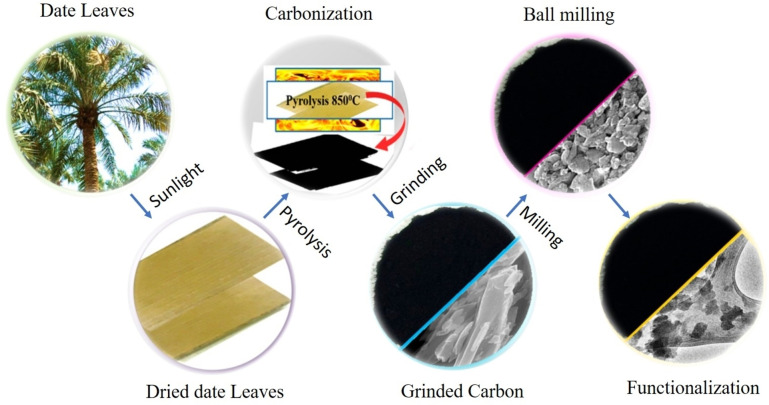
Diagram illustrating the steps in synthesizing the functionalized nanoparticle date carbon.^[6]^ Reproduced with permission from reference 6, Haq B, Aziz MdA, Al Shehri D, et al. Nanomaterials. 2022;12(8):1245, Copyright 2022, MDPI.

Biomass‐based CDs offer a promising avenue for GEOR compared to traditional methods due to their unique properties. The optimal conditions for synthesizing biomass‐based CDs for GEOR involve using biomass as a renewable and cost‐effective carbon source, such as date leaves, guava leaves, or banana peels, to prevent environmental pollution and reduce synthesis costs.[^6^, ^10^, ^13^, ^14^] The synthesis process typically involves hydrothermal or microwave irradiation to convert the biomass into CDs with high carbon content, excellent superhydrophobic characteristics, and superior oil‐absorbing capabilities^[10]^ ^[14]^. Ball milling is an inexpensive method to produce nanomaterials. It is a top‐down technique that involves milling powdered material into nanoparticles with balls of different stiffness. The milling process kinetics rely on energy, ball type, size, speed, temperature, and duration.[^15^, ^16^] Conversely, pyrolysis represents a straightforward and widely used controlled thermochemical process for transforming waste materials or alternative biomass into valuable commodities. This method is frequently employed in producing biochar, charcoal, and biogas for diverse industrial purposes.[^17^, ^18^] The purification process includes filtering the reaction mixture to eliminate larger particles and impurities and then dialyzing the solution to enhance the purity of the carbon dots.^[19]^ This method offers a green and environmentally friendly approach to converting biomass waste into valuable products and demonstrates the potential for applications in EOR .^[20]^ Varshney et al. demonstrated the development of superhydrophobic cotton coated with CDs for selective oil separation, showcasing excellent oil absorbing capabilities and high separation efficiency.^[10]^ Additionally, Haq et al. explored CNTs derived from date leaves in GEOR processes, showing significant incremental oil recovery when used in smart water and green chemical flooding applications, schematically illustrated in Figure 1.^[6]^ These studies indicate that biomass‐based CDs can enhance oil recovery through improved separation, flooding techniques, and foam stability in challenging reservoir conditions.

### 2.2 Properties of CDs

#### 2.2.1 Structure and Size

From a structural perspective, CDs are a quite broad class of nanomaterials with various architectures and adjustable optical characteristics. Most of their morphology is quasi‐spherical, and their structure might be amorphous^[21]^ or graphitic.^[22]^ CDs, which were identified in the middle of the 2000 s[^22^, ^23^] are the most significant character in carbon nanoscience. They are typically smaller than 10 nm, which may allow for easy penetration and distribution within the complex network of pores and fractures in the reservoir.

#### 2.2.2 Fluorescence Properties

The optical properties of CDs can vary depending on the synthesis procedures used, resulting in different subtypes capable of emitting fluorescence at various wavelengths. They can emit blue,^[24]^ green,^[22]^ or red light,^[25]^ and their fluorescence may remain constant regardless of excitation wavelength,^[26]^ or be adjustable, meaning the emission peak shifts continuously based on the excitation wavelength.^[27]^ This makes multiplexing possible, allowing several CDs to track various parameters simultaneously. As CDs have a high fluorescence quantum yield,^[28]^ it is possible to detect strong signals even at low concentrations, which makes real‐time monitoring of fluid movements within the reservoir, allowing for better management of EOR processes. Furthermore, CDs exhibit excellent photostability^[29]^ for long‐term tracking and monitoring applications.

#### 2.2.3 Surface Functionalization

Different chemical groups can be used to functionalize the surface of CDs. They can be hydrophilic or hydrophobic,[^30^, ^31^] depending on the specific surface structures. Surface functionalization of CDs with hydrophobic groups like alkyl chains, aromatic rings, or specific oil‐soluble molecules can enhance their affinity for oil, enabling improved tracking and visualization of oil movement within reservoirs.[^32^, ^33^] Various methods, such as click chemistry, have been employed to effectively engineer the surface functionalization of CDs, allowing for precise control over the attachment of specific functionalities like fluorescent molecules.^[34]^ Additionally, altering the surface functional groups of semiconductor materials like tin dioxide (SnO_2_) has been shown to enhance their selectivity towards specific gases, indicating the potential for improving the selectivity of CD‐doped SnO_2_ towards ethanol by adjusting the carboxyl functional group content on the CD surface.^[35]^ This comprehensive approach to surface functionalization opens new possibilities for using CDs in oil tracking applications within reservoirs. Functionalizing CDs with hydrophilic groups^[36]^ such as carboxylates, amines, or hydroxyl groups can enhance their interaction with rock matrices, improving their adhesion to rock surfaces and enabling them to serve as probes for mapping reservoir heterogeneity and identifying fracture networks. Studies have shown that surface functionalization of CDs through covalent or non‐covalent modifications can significantly impact their properties, making them more versatile for specific applications.^[32]^

#### 2.2.4 Biocompatibility

CDs exhibit excellent biocompatibility, low cytotoxicity, and stable chemical properties, making them ideal for various forensics, environmental science, and medicine applications, offering innovative solutions for detection, diagnosis, and treatment.[^9^, ^37^] The sustainable nature of CDs, derived from plant‐based and animal‐based precursors, further enhances their appeal by providing environmentally friendly and renewable sources for synthesis, ensuring minimal environmental impact.^[9]^ The potential of these nanostructures unveils innovations that not only push the boundaries of detection and diagnostics but also promise to reshape our approach to solving complex problems in new areas like minimizing concerns about environmental impact and potential damage to reservoir formations.

#### 2.2.5 Chemical Stability

CDs exhibit remarkable chemical stability, making them resilient to harsh reservoir conditions like high temperatures, pressures, and salinity.[^12^, ^38^, ^39^] Studies have shown that water‐soluble CDs (CDs−W) remain stable even at 80 °C, withstanding salinity levels of up to 200,000 mg⋅L^−1^ and Ca^2+^ concentrations of 1000 mg⋅L^−1^.^[38]^ Additionally, introducing CDs into styrene‐divinylbenzene copolymer microspheres significantly enhances their compressive strength to withstand closure pressures of up to 80 MPa with minimal deformation.^[39]^ Furthermore, the inert nature of CDs, their stability in aqueous solutions regardless of pH or salinity, and their ability to be quickly recovered from porous media without causing damage to the reservoir make them ideal for use as conservative tracers in reservoir characterization for carbon capture and storage (CCS) applications.^[12]^

### 2.3 Mechanisms in EOR

#### 2.3.1 Interfacial Tension Reduction

Water does not spontaneously imbibe into the porous medium in oil‐wet carbonate systems because the oil is firmly bound to the rock surface via capillarity. Surfactants can reduce capillarity‐based interfacial tension force that holds oil in place, and as a result, oil droplets pass down into the pores more easily and combine with oil downstream to create an oil bank.[^40^, ^41^] Using biomass‐based CDs in GEOR can potentially reduce interfacial tension between water and oil phases. CDs play a crucial role in reducing the interfacial tension between oil and water, facilitating the movement of trapped oil droplets through porous rock formations.^[42]^ Combining CDs with surfactants like hyperbranched polyglycerol (HPG), the interfacial tension can be significantly lower, observed in experiments where the interfacial tension dropped to 0.9 mN/m from higher values when using CDs and HPG together.^[42]^ Additionally, the introduction of CDs in styrene‐divinylbenzene copolymer microspheres develops ultra‐low‐density proppants with high strength and anti‐deformation properties, showcasing a peak pressure of 41.5 N and minimal axial deflection of 0.090 mm.^[43]^ These findings highlight the potential of CDs in enhancing oil extraction processes by reducing capillary forces and aiding in the mobilization of oil from reservoirs through the reduction of interfacial tension.

#### 2.3.2 Wettability Alteration

**Figure 2:**
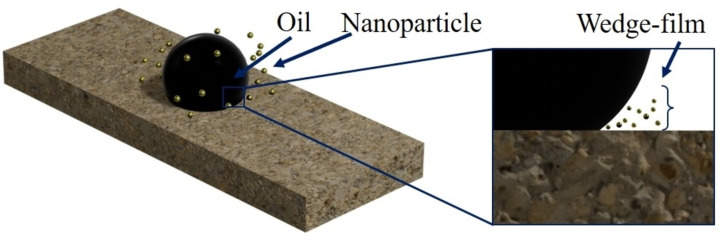
Nanoparticle assembling in wedge film causes structural disjoining pressure.^[45]^ Reproduced with permission from reference 45, Jafari Daghlian Sofla S, Anne James L, Zhang Y., E3S Web of Conferences. 2019;89:03004., Copyright 2019, EDP Sciences.

**Figure 3:**
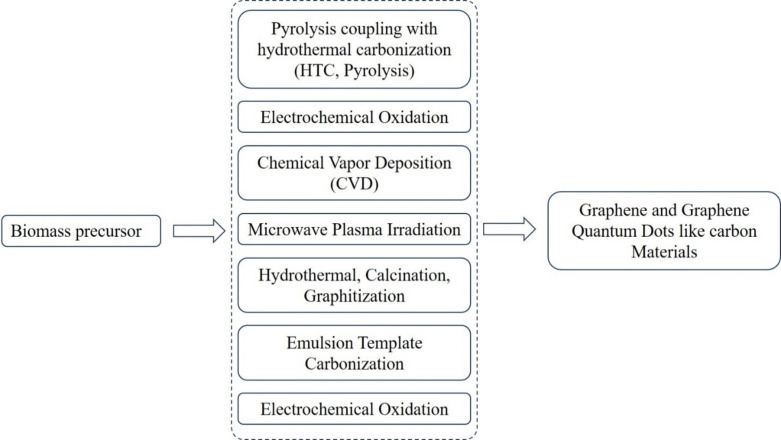
Synthesis of graphene via various routes.^[146]^ Reproduced with permission from reference 146, Saha JK, Dutta A., Waste Biomass Valorization. 2022;13(3):1385‐1429., Copyright 2022, Springer Nature.

CDs can alter the wettability of reservoir rock surfaces, transitioning them from oil‐wet or mixed‐wet to water‐wet conditions, as indicated by studies on chelating agents.^[44]^ The interaction between CDs and the rock surface changes electrostatic properties, affecting the repulsion or attraction of charged particles like nanoparticles, which play a crucial role in wettability alteration.^[45]^ Many factors affect the wettability of substrates when using nanoparticle‐fluids, such as size and concentration of the nanoparticles, initial contact angle,^[46]^ particle charge, surface wettability of the nanoparticles,^[47]^ charge and roughness of the substrate surface, concentration of stabilizers, type, and concentrations of ions in the nanoparticle‐fluids, bulk pressure, temperature, etc. By examining the nanoparticles′ self‐layering, Kondiparty et al.^[48]^ concluded that the three‐phase contact line naturally diminishes to establish an equilibrium situation. As seen in Figure 2, this ordering in the wedge‐like region results in an additional pressure in the film compared to the bulk solution and separates the oil drop from the surface. The term “structural disjoining pressure” refers to this pressure (Figure 3).

Sefiane et al.^[49]^ highlighted that alterations in the contact angle of oil, water, and rock surfaces may result from a combination of “structural disjoining pressure” and nanoparticle adsorption on the rock surface. Kondiparty et al.^[46]^ observed that increasing nanoparticle concentration enhances both structural disjoining pressure and spreading rate of the nanoparticles‐fluid. Additionally, they noted a decrease in the spreading rate of nanoparticles‐fluid as the drop volume decreased. Wang et al.^[47]^ explored the impact of particle charge and nanoparticle surface wettability on oil drop detachment from a surface through molecular dynamic simulations. They concluded that highly charged hydrophobic nanoparticles exhibit superior performance in oil detachment. Lim et al.^[50]^ illustrated that an increase in temperature and substrate hydrophilicity accelerates the detachment of oil droplets. This comprehensive approach to wettability alteration mechanisms highlights the potential of carbon dots in improving enhanced oil recovery strategies by promoting water‐wet conditions on reservoir rock surfaces.

#### 2.3.3 Improved Sweep Efficiency

Nanoparticles have shown promise in stabilizing oil and water emulsions, preventing droplet coalescence. Hu at al.^[51]^ established that core permeability decreases from 834.86×10^−3^ μm^2^ to 219.34×10^−3^ μm^2^, and the pickering emulsion‘s incremental oil recovery rises from 12.36 % to 17.39 % in core flooding trials. With the help of Fe_3_O_4_@PDA@Si nanoparticles, this novel Pickering emulsion flooding system stabilizes, providing an EOR alternative. This stabilization effect is achieved through the synergistic action of cationic surfactants and CDs, ensuring the dispersion of the oil phase and facilitating more accessible transportation through reservoirs.^[52]^ Additionally, nanofluids containing CDs can enhance sweep efficiency by improving penetration into porous rock structures, as demonstrated by the increased oil recovery and improved displacement efficiency observed in experiments.^[53]^ The combination of CDs with surfactants in emulsions stabilizes the system and also allows for the controlled switch between stable and unstable modes, offering versatile applications in various industries, including enhanced oil recovery processes.^[52]^

#### 2.3.4 Chemical Interactions

The chemical interactions between CDs and oil molecules influence the oil recovery by enhancing foam stability and mobility control in high salinity brine conditions. Sakthivel et al.^[54]^ demonstrated that adding CDs in trace amounts (5‐10 ppm) led to a significant improvement of 60–70 % in foam stability, attributed to reducing the drainage rate of the lamellae and delaying the bubble rupturing point. This improvement in foam stability contributed to improved foamability, morphology, strength, and overall stability, leading to enhanced inflow performance and added oil recovery. The multifaceted interrelationship between CDs, surfactants, and oil molecules positively impacts the efficiency of foams in controlling fluid channeling and fingering in high permeability reservoirs.

#### 2.3.5 Viscosity Reduction

One of the main variables affecting the use of polyacrylamide (PAM) in EOR is viscosity. Alhaji Haruna et al.^[55]^ reported a comprehensive study of the stability and interactions of PAM, CDs, and PAM/CDs. They observed that CDs lower PAM solution viscosity contrary to Einstein–Batchelor law. The decrease in viscosity can be attributed to the disintegration of the PAM polymer caused by deamination within the polymer‘s repeating unit, resulting in the generation of polyacrylic acid (PAA) and ammonia (NH_3_). Incorporation of CDs in the composites leads to a significant presence of oxygen‐containing groups in the CDs, which interact with the PAM material through a nucleophilic process, compelling the release of –NH_2_ groups from the PAM repeating unit, thereby fostering the preparation of PAA and NH_3_. These patterns persist and weaken the polymer structure, causing deterioration at the macromolecular level and consequent decline in PAM's flexibility and toughness, resulting in viscosity reduction, potentially improving oil recovery mechanisms through enhanced flow properties.^[55]^

### 2.4 Benefits of using CDs

#### 2.4.1 Cost‐Effective

Studies have shown that CDs, derived from biomass like guava leaves or pencil leads, exhibit excellent properties for GEOR applications, such as high colloidal stability, superior oil absorption capacity, and the ability to improve foam stability in high salinity brine.[^6^, ^54^, ^56^, ^57^] These nanomaterials can enhance oil recovery by increasing microscopic sweep efficiencies, boosting tertiary oil production while being environmentally friendly. The use of CDs in GEOR processes provides a sustainable and green alternative. It offers cost‐effective solutions for the industry by enabling the development of smart water floods and green chemical agents for efficient oil production and recovery.

#### 2.4.2 Environmental Impact

CDs offer significant environmental benefits in EOR compared to traditional methods. Carbon nanoparticles contribute to GEOR processes, enhancing oil production sustainably and economically.^[6]^ Additionally, CDs have been utilized in foam systems to improve stability and efficiency in EOR applications, leading to enhanced inflow performance and added oil recovery.^[54]^ Using CDs in EOR enhances oil recovery and reduces the environmental impact by offering green alternatives to traditional methods, making them a promising solution for the oil and gas industry regarding sustainability and environmental conservation.

#### 2.4.3 Enhanced Efficiency

By incorporating carbon nanomaterials like CDs, EOR processes can achieve superior oil recovery rates while being environmentally friendly.^[6]^ CDs in EOR have shown promising results in reducing interfacial tension and altering wettability, leading to increased oil recovery from carbonate reservoirs.^[58]^ Furthermore, developing smart superhydrophobic surfaces using CDs has demonstrated excellent oil separation capabilities, showcasing their potential for efficient oil spill cleanup applications.^[10]^ Overall, using CDs in EOR presents a modern and practical approach to achieving enhanced efficiency in oil recovery processes.

#### 2.4.4 Enhanced Monitoring

For 60 years, tracers have been widely used in the oil and gas sector. Among the most significant and common uses for tracers are water erosion processes, hydrogeological research, geothermal studies, and well connection determination.[^59^, ^60^] Compared to conventional tracers, CDs offer significant advantages for EOR monitoring. The stability of CDs in the presence of metallic ions, high salt concentrations, pH changes, and their behavior in porous media under static and dynamic circumstances have all been well studied in laboratory settings.[^61^, ^62^] Rosales et al. ^[56]^ established that their work opens the door for the industry to adopt CDs, especially in areas where complex injector and producer well arrangements necessitate the use of several tracers to provide a complete system description. Overall, the application of CDs in EOR broadens the monitoring field and enhances the overall effectiveness of monitoring processes in the oil and gas industry.

## 3 CNTs and their Derivatives in Enhanced oil Recovery

The two primary types of CNTs are single‐walled (SW) and multi‐walled (MW), depending on how many graphene sheets make up each cylindrical tube. CNTs are one‐dimensional cylindrical nanomaterials with exceptional mechanical, thermal, and electrical properties.^[63]^ Their high aspect ratio and nanoscale dimensions make them ideal for enhancing oil displacement in EOR applications. CNTs can be incorporated into fluids injected into reservoirs to penetrate deep into porous rock formations, reducing interfacial tension and improving oil mobility.^[63]^ Moreover, CNTs can act as reinforcing agents in polymer‐based EOR formulations, boosting stability and performance under reservoir conditions.^[63]^ The synthesis methods for CNTs include laser ablation, electric arc discharge, chemical vapor deposition, and other techniques, offering a wide range of possibilities for tailoring CNT properties to suit specific EOR requirements .^[64]^ The utilization of these nanoparticles in GEOR applications has shown promising results in reducing interfacial tension, altering wettability, and improving oil recovery rates .[^65^, ^66^] These versatile nanoparticles are promising to revolutionize GEOR strategies through their unique characteristics and applications in the oil and gas industry.

### 3.1 Synthesis of CNTs

The key steps in synthesizing eco‐friendly biomass‐based CNTs involve using natural and renewable precursors. Various natural hydrocarbon precursors like plant extracts, essential oils, and agricultural bio‐wastes are used to fabricate CNTs in an environmentally friendly and cost‐effective manner.^[67]^

Microwave (MW) methods for producing CNTs offer cost reduction, fast reaction times, easy purification, and eco‐friendliness.^[68]^ MWCNTs can be quickly synthesized on a graphite surface using ferrocene as a catalyst, showcasing the advantages of low temperature and shorter reaction times with MW technique.^[69]^ Purification and functionalization of CNTs are necessary for further applications, with MW‐assisted protocols being advantageous due to the rapid coupling of carbonaceous materials. Various purification techniques, including thermal, chemical, and sonication treatments, are commonly used,[^70^, ^71^] with MW irradiation‐based purification significantly enhancing properties like thermal conductivity and diffusivity of MWCNTs.^[72]^ Examples of CNTs prepared using MW‐assisted chemical vapor deposition and MW pyrolysis techniques are provided in Table 1, demonstrating benefits such as uniform heating, higher yields, and improved mass transfer of volatiles.

**Table 1:** Several examples of CNTs made from biomass under MW irradiation.

CNTs	Catalysts	Synthesis method	Biomass	Ref
MWCNTs 50‐200 nm	Nikel	MW‐chemical vapor deposition	Rice Husk	^73^
MWCNTs 17‐100 nm	Ferrocene		Oat hulls, hazelnut hulls, wheat straw, and rapeseed cake	^74^
MWCNTs 50 nm	Nikel		Pine nutshell	^75^
MWCNTs 50‐100 nm	Mineral matter in char particles from biomass	MW pyrolysis at low temperature 600^0^C	Gumwood	^76^
MWCNTs 50‐100 nm	‐		Palm kernel shell cellulose	^77^

A process using vapor‐assisted ozone treatment was reported for functionalizing CNTs to introduce oxygen groups, mainly carboxyl groups at near ambient temperature.^[78]^ The treatment was done with compressed air as the carrier gas, providing an environmentally friendly functionalization procedure. Additionally, CNT suspension was treated with ozone to create ultra‐high‐performance concrete/CNT composites.^[79]^

Functionalization of SWCNTs was achieved using poly (ethylene glycol) grafted polymers in aqueous media via Diels–Alder click reaction under ultrasonic irradiation at room temperature.^[80]^ Ultrasonic irradiation was used on carboxylated MWCNTs‐COOH with thiamine to produce nanocomposites with improved properties.^[81]^ While ultrasound‐assisted techniques have been used for CNT surface modification and dispersion, the quality of CNTs produced this way is not superior to those obtained by traditional methods. It is unlikely that ultrasound‐assisted CNT production will be used industrially without significant improvements and optimization.^[82]^ These biomass‐derived carbon nanotubes can be tailored to address the unique challenges and demands of different oil fields, contributing to integrating nanotechnology in the hydrocarbon industry to combat climate change.

### 3.2 Properties and Characteristics

#### 3.2.1 Structure

CNTs possess unique structural properties that make them ideal for EOR applications. These properties include high conductivity, mechanical strength, and superior aspect ratios, making them highly suitable for subsurface applications.^[83]^ Additionally, CNTs exhibit superhydrophobicity, superoleophilicity, high surface area, low density, chemical stability, excellent mechanical properties, and large pore volume, which are advantageous for oil‐water separation processes.^[84]^ Furthermore, CNTs have been shown to reduce the interfacial tension between oil/water, form in‐situ surfactants, and lower capillary pressure within small pores, thereby enhancing crude oil recovery.^[83]^ The use of modified multi‐walled CNTs as additives in polyacrylamide solutions has also been explored to increase the oil recovery factor, highlighting the versatility and potential of CNTs in EOR applications.

#### 3.2.2 Mechanical Strength

The mechanical strength of CNTs plays a crucial role in their potential application in EOR. Studies have shown that CNTs, especially MWCNTs, exhibit high mechanical strength, making them suitable for EOR processes.[^83^, ^84^] Incorporating CNTs into composite materials, such as silicone foams, enhances the matrix‘s elastic modulus and collapses stress, improving mechanical stability under compression loads.^[84]^ Additionally, the hydrophobic and oleophilic behavior of CNTs contributes to their high mechanical strength, making them effective in oil spill recovery applications.^[85]^ The unique characteristics of CNTs, including their high aspect ratios and superior mechanical properties, make them promising candidates for enhancing oil recovery processes and addressing the challenges associated with hydrocarbon dependency and carbon sequestration.[^83^, ^86^]

#### 3.2.3 Electrical and Thermal Conductivity

CNTs exhibit exceptional electrical and thermal conductivity properties, making them valuable in EOR applications. CNTs, specifically SWCNTs, and MWCNTs, can enhance the thermal conductivity of nanoparticle‐oil suspensions, with SWCNTs demonstrating higher thermal conductivity than MWCNTs. Additionally, incorporating hydroxyl‐functionalized MWCNTs in polyurethane nanocomposite bioplastics significantly enhances electrical conductivity, mechanical properties, and thermal stability, offering a sustainable solution for EOR and carbon sequestration projects.^[87]^ The use of CNTs in composite materials with silicone foams has also shown promising results in oil spill recovery applications, highlighting their hydrophobic and oleophilic behavior, mechanical strength, and high oil selectivity, which are crucial for efficient cleanup technologies.^[85]^ By leveraging these properties, CNTs can play a pivotal role in addressing the challenges of EOR while promoting a carbon‐negative future.

#### 3.2.4 High Surface Area

The high surface area property of CNTs can be optimized for EOR in specific geological formations. CNTs, particularly MWCNTs, can be modified and used as additives to EOR by reducing interfacial tension and altering wettability.^[88]^ Additionally, applying CNTs in combination with green surfactants like alkyl polyglucoside (APG) has demonstrated promising results in recovering residual oil within rock pores, leading to significant incremental oil production.^[86]^ By leveraging the unique properties of carbon nanotubes, such as their high surface area, researchers can optimize their application for EOR in specific geological formations.

#### 3.2.5 Hydrophobicity

CNTs exhibit remarkable hydrophobic properties, making them ideal for oil‐water separation.^[84]^ These properties include superhydrophobicity and superoleophobicity, allowing CNTs to.^[89]^ Moreover, CNTs possess high sorption capacities, with absorption capabilities ranging from 15 to 50 times their weight, making them efficient in oil recovery applications.^[84]^ The hydrophobic nature of CNTs is also leveraged in the development of hydrophobic membranes for oil/water separation, where carbon nanotube/ carbon nanofiber hybrid membranes show high separation efficiency (80 %‐98 %) and recyclability, making them practical for purifying oil‐contaminated water.^[90]^

#### 3.2.6 Chemical Stability

CNTs have shown promising potential in enhancing oil recovery due to their unique properties, such as reducing interfacial tension and altering wettability.[^66^, ^91^] The functionalized MWCNTs can effectively reduce interfacial tension between nanofluids and oil, improving recovery rates.^[91]^ In addition, the stability of nanofluids containing CNTs has been confirmed, with tests showing stability for up to 10 days in varying salinity conditions.^[91]^ Furthermore, the application of modified MWCNTs as additives in polyacrylamide solutions has been explored to enhance oil recovery factors. These findings highlight the chemical stability and effectiveness of CNTs in improving oil recovery processes, making them a promising candidate for future applications in the oil industry.

#### 3.2.7 Nanoparticles Adsorption

Nanoparticles, such as silica, CNTs, and TiO_2_, alter wettability and enhance fluid displacement efficiency in various subsurface applications. These nanoparticles can modify the rock surface, promoting water‐wet conditions favorable for CO_2_ trapping.^[92]^ Additionally, in EOR, nanoparticles like MWCNTs, SiO_2_, and Al_2_O_3_, when combined with surfactants, reduce fingering effects, increase recovery factors, and alter oil‐water interfacial tension.^[93]^ Furthermore, nanofluids aid in opening micropores, increasing specific surface area, pore volume, and diameter in coalbed methane reservoirs, enhancing water wettability and reducing water lock damage.^[94]^ The adsorption of nanoparticles onto rock surfaces can selectively plug smaller pores, diverting displacing fluids into larger, oil‐rich pores, thereby enhancing sweep efficiency and reducing bypassing of oil‐rich zones, as observed in various studies on nanofluids′ impact on fluid displacement and wettability alteration.[^94^, ^95^]

### 3.3 Mechanisms in EOR

#### 3.3.1 Viscosity Reduction

Reducing heavy oil viscosity involves physical and chemical methods. Physical techniques like heating and dilution are common but costly. Chemical methods use agents to form oil‐in‐water (O/W) emulsions, reducing viscosity through intermolecular forces.[^96^, ^97^] Lower viscosity leads to better O/W emulsion flow, reduced water bypassing, and enhanced oil displacement efficiency.[^98^, ^99^] Viscosity‐reducing agents have hydrophilic and lipophilic groups for migrating to oil‐water interface, improving water flooding efficiency. Viscosity reducers emulsify at oil‐water interface, but viscosity quickly reverts after destabilization.[^99^, ^100^] Emulsification efficiency of agents is crucial for oil recovery.[^101^, ^102^] Nanoparticles have been studied to enhance oil‐water interface stability and emulsification effects.^[103]^ Nanoparticles with different properties form different emulsions, affecting viscosity reduction.^[104]^

CNTs with amino, carboxyl, and hydroxyl groups were fabricated by Hua et al.^[105]^ as a means of reducing nano viscosity. The structure‐property link of CNTs in lowering the viscosity of crude oil was examined through experiments. The interfacial behavior of CNTs at the oil‐water contact was investigated using molecular dynamics simulations. This work offers design guidelines for the molecular structure of future high‐efficiency nano‐viscosity reducers. MWCNTs are used as additives in polyacrylamide solutions to increase the oil recovery factor. These nanotubes can reduce the interfacial tension between oil and water, improving oil recovery by lowering capillary pressure within small pores.^[83]^ Applying CNTs and green surfactants displace oil effectively due to their unique phase‐behavior properties, ultimately leading to increased oil recovery rates.^[86]^ Furthermore, simulations have demonstrated that nanomaterials like CNTs can increase oil mobility, lower interfacial tension, and improve oil recovery, especially at lower flow rates.^[106]^

#### 3.3.2 Interfacial Tension Reduction

CNTs have shown promise in enhancing oil recovery due to their ability to reduce interfacial tension and alter reservoir rock wettability, ultimately increasing oil. CNTs can reduce the capillary pressure within small pores when dispersed in base fluids to form nanofluid, facilitating crude oil recovery^[83]^ . Additionally, CNTs can be imbibed with surfactants and coated with wax, which is triggered to disgorge the surfactants upon contact with oil or exposure to specific temperatures, leading to improved oil recovery by reducing interfacial tension and changing wettability. The synergistic effects of combining CNTs with surfactants have been observed to reduce interfacial tension further, alter rock wettability, stabilize foams or emulsions, and enhance EOR potential .^[107]^ Overall, the unique properties of CNTs make them valuable in advancing EOR techniques and addressing the challenges of hydrocarbon recovery and carbon sequestration.^[83]^

CNTs also possess properties like superhydrophobicity, superoleophilicity, high surface area, and excellent mechanical properties, making them ideal for reducing interfacial tension between oil and water.^[108]^ When CNTs are used to deliver surfactants to the oil‐water interface, they can adsorb surfactant molecules and decrease in interfacial tension.^[109]^ Additionally, CNT membranes can facilitate fast water transport due to their structure, although a pressure loss at the pore hinders overall transport efficiency.^[110]^ Furthermore, the effects of CNTs on interfacial tension were studied by synthesizing MWCNTs and conducting experiments to understand their impact, showing a reduction in surface tension with varying concentrations of CNTs.^[111]^ CNTs′ properties and interactions with surfactants are crucial in reducing the interfacial tension between oil and water, offering promising applications in oil‐water separation processes.

#### 3.3.3 Wettability Alteration

CNTs can modify the wettability of reservoir rocks through various mechanisms.^[112]^ Oxygenated functional groups on CNT arrays can lead to superhydrophobic behavior, making them extremely water‐repellent, while high surface concentrations of these groups result in extreme hydrophilicity. Additionally, ion‐beam modification methods can control the wettability of CNT coatings, making them hydrophobic or hydrophilic to different types of liquids.^[113]^ When considering the alteration of solid surfaces due to nanoparticles in fluids, it is crucial to account for the complex interactions between nanoparticles, diverse ions in formation water, and surface‐active components in the oil phase, which can impact the electrostatic properties and wettability of the reservoir rock.^[45]^ Moreover, the wettability of reservoir rocks can be altered by divalent ions at high temperatures, with sulfate ions showing a greater effect in the presence of magnesium and calcium ions.^[114]^

#### 3.3.4 Formation of Stable Emulsions

CNTs can act as 1D emulsion stabilizers, replacing traditional surfactants, and exhibit amphipathic properties that enable the formation of water‐in‐oil emulsions.^[115]^ Additionally, CNT/MgO nanocomposites have been shown to reduce interfacial tension, alter wettability from oil‐wet to water‐wet conditions, and enhance oil recovery factors significantly.^[116]^ Furthermore, modified MWCNTs have been explored as additives to polyacrylamide solutions to increase oil recovery factors. The application of carbon nanomaterials like CNTs in green EOR processes has also been investigated, showcasing their potential to.^[6]^ CNTs demonstrate promise in EOR by stabilizing emulsions, reducing interfacial tension, altering wettability, and enhancing oil recovery factors, thus contributing to more efficient and environmentally friendly oil extraction processes.

### 3.4 Benefits of using CNTs

Due to their special qualities, CNTs have become increasingly popular in EOR, which aims to collect more crude oil from an oil field. Some advantages of employing CNTs in EOR include:

#### 3.4.1 Improved oil Displacement Efficiency

CNTs enhance oil recovery by altering the wettability of reservoir rocks from oil‐wet to water‐wet, thus aiding in the movement of oil toward production wells.^[117]^ They have a high surface area, which can significantly improve the interaction with oil droplets, ultimately enhancing the efficiency of oil displacement processes.^[118]^ The unique physical and chemical properties of CNTs, including their ability to be oxidized to enhance dispersion in polar solvents, contribute to their effectiveness in oil recovery operations .^[119]^ By utilizing CNTs in engineered water‐surfactant systems, achieving a water‐wet state in carbonate reservoirs can increase oil recovery rates through improved solubility and interfacial activity .^[120]^

#### 3.4.2 Increased Mobility Control by Viscosity Modification

Studies have highlighted the effectiveness of CNT‐based systems in improving foam stability and mobility reduction during gas injection for EOR operations^[121]^ . By dispersing CNTs in base fluids to form nanofluids, the interfacial tension between oil and water can be reduced, aiding in crude oil recovery.^[83]^ Additionally, using CNTs combined with surfactants like alpha olefin sulfonate (AOS) has significantly increased foam stability and mobility control, outperforming traditional systems like AOS‐silica foaming systems.^[121]^

#### 3.4.3 Enhanced Sweep Efficiency

CNTs in EOR processes offer significant benefits in enhancing sweep efficiency. CNTs, due to their unique properties like reducing interfacial tension between oil/water, forming in‐situ surfactants, and altering wettability, contribute to increased oil recovery by improving oil displacement within reservoir pores.[^83^, ^86^, ^88^] By dispersing CNTs in base fluids to create nanofluids, the mobility of injected CO_2_ can be reduced, forming a uniform front and enhancing carbon storage potential in the reservoir.^[83]^ Applying CNTs with green surfactants has shown promising results in recovering residual oil within rock pores, with formulations containing CNTs and green surfactants producing significant incremental oil after water flooding.^[86]^ These findings highlight the potential of CNTs to optimize sweep efficiency and improve oil recovery in EOR processes, paving the way for more effective and sustainable reservoir management strategies.

#### 3.4.4 Environmental Benefits

CNTs offer significant environmental benefits when utilized in EOR processes. Using functionalized CNTs in EOR processes can lead to the fabrication of composite materials with improved mechanical, chemical, and thermal properties, enhancing the overall efficiency of environmental remediation techniques.^[122]^ These advancements highlight the crucial role of CNTs in promoting a more sustainable and environmentally friendly approach to EOR processes. Integrating CNTs in EOR technologies offers promising improvements in efficiency, cost‐effectiveness, and environmental impact, making them a valuable tool in modern oil recovery processes.

## 4 Graphene and its Derivatives in Enhanced Oil Recovery

Graphene, a remarkable two‐dimensional carbon‐based material with a unique honeycomb lattice structure, exhibits exceptional properties like high surface area, mechanical strength, thermal conductivity, and chemical stability.[^123^, ^124^, ^125^] These characteristics make graphene a promising candidate for various applications, including EOR in the petroleum industry. Graphene‐based materials can be effectively utilized as additives in drilling fluids or as coatings on wellbore surfaces to prevent formation damage, enhance fluid flow, and improve EOR processes.^[126]^ Moreover, graphene‐based membranes have demonstrated significant potential for selective oil‐water separation, aiding in the purification of produced fluids and reducing environmental impact by efficiently separating oil from water mixtures.^[126]^ The versatility and effectiveness of graphene in EOR applications underscore its importance in advancing sustainable and efficient practices in the oil and gas sector, showcasing its potential for revolutionizing processes in the industry.^[123]^

### 4.1 Synthesis of Graphene and its Derivatives

Numerous efforts have been dedicated to the production of high‐quality large‐area graphene. The initial technique, involving the mechanical exfoliation of graphene from highly oriented pyrolytic graphite using Scotch tape, results in good quality but μm‐sized graphene.^[127]^ Alternative methods, like epitaxial graphene grown on single crystalline SiC substrates[^128^, ^129^] or transition metals,[^130^, ^131^] can produce larger graphene domains. Recently, high‐quality monolayer or bilayer graphene has been successfully synthesized through chemical vapor deposition (CVD) of CH_4_ or C_2_H_2_ gases on copper or nickel substrates.[^132^, ^133^] The CVD technique is currently widely utilized and shows significant potential for the large‐scale fabrication of high‐quality films.[^134^, ^135^, ^136^] Furthermore, simpler, cost‐effective, and safer techniques for graphene growth have been developed, employing solid carbon sources such as polymers, SiC, and amorphous carbon.[^137^, ^138^, ^139^] An oxygen‐free environment is essential during graphene growth to prevent carbon oxidation and catalyst surface oxidation at elevated temperatures. Growing graphene in a non‐vacuum setting could lead to novel applications. Recent studies have demonstrated the growth of graphene at the silicon dioxide and metal catalysts interface, with the Pt‐based catalyst approach proving beneficial, as discussed in a specific reference.[^140^, ^141^] Additionally, a catalyst‐free approach was introduced in another reference.^[142]^ Multiple synthesis pathways exist for graphene, including mechanical and chemical exfoliation, chemical vapor deposition, pyrolysis, and epitaxial methods for producing graphene films.^[143]^

Consideration of biomass‐based graphene and its derivatives synthesis holds significant promise for various applications, including EOR. Biomass waste, such as agricultural residues and lignin, can be converted into graphene and graphene‐like materials through pyrolysis and chemical exfoliation .[^144^, ^145^] By harnessing the renewable nature of biomass waste and the versatile synthesis methods available, biomass‐based graphene and its derivatives can contribute to more sustainable and efficient EOR practices.

### 4.2 Properties and Characteristics

#### 4.2.1 Structural Properties

Graphene oxide (GO) possesses amphiphilic properties, high reactivity, and thermal stability, making it a promising material for EOR applications.[^147^, ^148^] Incorporating GO into hydrolyzed polyacrylamide (HPAM) solutions significantly enhances the interfacial properties at the oil‐water interface, reducing interfacial tension and contact angle while improving mobility behavior and oil recovery efficiency.^[147]^ Additionally, the interaction of GO with polymer solutions can double the viscosity, indicating its ability to modify the rheological properties of the fluid system, which is crucial for EOR processes.^[149]^ Furthermore, the synthesis of a GO‐SiO_2_ nanocomposite has demonstrated the potential to alter reservoir wettability effectively, transitioning from oil‐wet to water‐wet conditions, especially under high‐temperature and high‐salinity environments, showcasing its adaptability to harsh EOR conditions.^[7]^

#### 4.2.2 High Surface Area

GO and reduced GO (rGO) exhibit high surface area properties and unique characteristics that make them valuable in various applications, including enhanced oil recovery. GO‐based materials are known for their amphiphilic properties, thermal stability, and reactivity, making them suitable for emulsification processes.^[148]^ rGO has been extensively explored in energy‐related technologies due to its high surface area, tunable conductivity, and ease of modification, showing promise in fuel cells, supercapacitors, and lithium‐ion batteries.^[150]^ The preparation of activated reduced GO (arGO) into a 3D analog of defect‐rich GO has been reported, demonstrating a high surface area suitable for sorption applications, such as U(VI) removal, due to its hydrophilic nature and microporous structure.^[151]^ These properties of GO and rGO, including high surface area and unique chemical characteristics, contribute to their effectiveness in enhanced oil recovery processes, as seen in the development of nanohybrids like GO/PAM and CNT/PAM for improved oil recovery through wettability alteration and interfacial tension reduction mechanisms.^[152]^

#### 4.2.3 Mechanical Strength

The mechanical properties of graphene and its derivatives enhance oil recovery efficiency in complex reservoirs. Graphene nanoparticles have been shown to improve oil recovery factors by influencing reservoir properties such as wettability alteration, interfacial tension reduction, and stabilization of drilling fluids.[^153^, ^154^] The unique 2D structures and excellent physical and chemical properties of graphene make it a promising candidate for various applications in the oil and gas industry, including CEOR and profile control.^[155]^ Graphene nanoparticles can decrease the dynamic viscosity of oil, leading to improved mobility of the reservoir and increased oil recovery rates. The adsorption layers formed by nanoparticles on sandstone surfaces significantly alter wettability and interfacial tension, further enhancing oil recovery efforts in high‐complexity reservoirs.^[154]^

#### 4.2.4 Thermal Conductivity

The addition of graphene nanoparticles leads to a significant decrease in the dynamic viscosity of oil, with reductions ranging from 10 % to 60 % depending on the concentration and temperature.^[156]^ Furthermore, graphene‘s exceptional thermal conductivity makes it a valuable material for improving the recovery factor of heavy oil reservoirs through aqua thermolysis reactions.^[156]^ The high thermal conductivity of graphene and its derivatives has been leveraged in developing thermally conductive polymer composites, showcasing their potential in enhancing the thermal properties of materials used in oil recovery processes.^[157]^ This collective evidence underscores the importance of graphene‐based materials in optimizing oil recovery strategies in challenging reservoir conditions.

#### 4.2.5 Chemical Stability

Aboahmed et al.^[158]^ studies have shown that the stability of GO enhanced polymer (GOeP) hybrids is influenced by factors such as temperature, salinity, and the presence of Mg^2+^ ions, with salinity and Mg^2+^ concentration significantly impacting long‐term stability. Using nanofluids containing graphene quantum dots nanoparticles has demonstrated improved bulk foam stability in foam EOR processes, leading to enhanced oil sweep efficiency and incremental oil recovery in various oil‐wet porous media^[158]^. The amphiphilic properties and high reactivity of GO make it suitable for emulsification processes in EOR applications.^[148]^ Graphene and its derivatives show great potential in EOR due to their unique structures and excellent physical and chemical properties, making them valuable for various oil and gas industry applications^[155]^ .

### 4.3 Mechanisms in EOR

#### 4.3.1 Wettability Alteration

Li and Firoozabadi postulated the potential for modifying the wettability near the wellbore using fluorine‐containing substances to transition from highly liquid‐wetting to moderately gas‐wetting conditions, aiming to prevent condensate buildup and pore blockage, thereby enhancing gas permeability and ultimately boosting the production of condensate gas reservoirs.[^159^, ^160^] Wang et al. illustrated a notable enhancement of about 30 % in both oil displacement efficiency and gas deliverability following treatment with fluorine‐based chemicals under elevated temperatures.^[161]^ These investigations highlight the potential benefits of gas‐wetting alteration in enhancing gas production and deliverability in gas‐condensate reservoirs. Utilizing functionalized nanomaterials holds promise in creating surfaces characterized by low surface energy and multi‐scale roughness. Functionalization techniques, such as using octadecyl‐functionalized crosslinked GO in ceramic membranes,^[162]^ can significantly impact the wettability of graphene‐based materials, reducing the underwater oil contact angle and increasing the water contact angle. This modification enhances the selective permeability of oil while maintaining high separation efficiency for water‐in‐oil emulsions. Chemical modification through covalent bonding can induce spin magnetism and catalytic activity in graphene, affecting its electronic structure and conductivity.^[163]^ These alterations improve the wettability properties of graphene and contribute to the development of advanced materials with enhanced oil recovery capabilities, showcasing the diverse applications and benefits of surface functionalization and chemical modification in graphene research.

#### 4.3.2 Interfacial Tension Reduction

The surface chemistry of graphene reduces interfacial tension in oil recovery applications. Wang et al. explore the modification of GO with different coupling agents like alkylamines, silane coupling agents, and haloalkanes to enhance its properties.^[164]^ The study reveals that alkylamines exhibit high stability when combined with GO, leading to the formation of surfactant‐like polymers with hydrophilic properties. Molecular dynamics simulations show that modified graphene oxide molecules at the oil‐water interface experience self‐aggregation, affecting the interfacial formation energy and interfacial tension at different temperatures. Understanding the binding energy and characteristics of these modified graphene oxide systems is essential for improving oil recovery efficiency through reduced interfacial tension.

#### 4.3.3 Viscosity Reduction

High‐viscosity oils might encounter challenges in permeating the porous geological structure of the reservoir,^[165]^ resulting in a decline in production rates and a rise in pressure gradient. One strategy to mitigate the viscosity of dense crude oil involves creating oil emulsions in water.^[166]^ The emulsification process can be attained through mechanical methodologies or by incorporating surfactants or emulsifying agents to uphold the stability of the emulsion. Another way is the addition of graphene nanoparticles to crude oil has been shown to impact its rheological properties significantly. The addition of graphene nanoparticles substantially reduces the viscosity of oil, enhancing its flow properties. This reduction improves the efficiency of oil transport and processing.^[167]^ Pakharukov et al.^[167]^ conclude that their experimental results demonstrate that varying the concentration of graphene nanoparticles affects the degree of viscosity reduction. Another research has demonstrated that graphene nanoparticles can lead to a notable decrease in viscosity, enhancing the fluidity of crude oil .^[168]^

### 4.4 Benefits of using Graphene and its Derivatives

Graphene and its derivatives, such as GO and rGO, offer several advantages for EOR due to their unique properties. Here are the benefits of using graphene and its derivatives in EOR.

#### 4.4.1 Superior Wettability Alteration

GO and rGO have shown significant potential in enhancing oil recovery through superior wettability alteration of reservoir rocks.^[169]^ A study by Sabbaghi et al. demonstrated the synthesis of a nanohybrid combining GO and silica (SiO_2_) for this purpose, showing that GO‐SiO_2_ nanocomposites can effectively change the wettability of rock surfaces from oil‐wet to water‐wet, crucial for improved oil recovery.^[7]^

#### 4.4.2 Improved Thermal Conductivity

Incorporating amphiphilic GO into hydrolyzed polyacrylamide (HPAM) solutions has improved interfacial properties, mobility control, and overall performance in EOR applications.^[147]^ The synthesis of Janus graphene nanosheets has demonstrated the ability to lower interfacial tension between brine and crude oil at simulated reservoir conditions, improving oil recovery efficient ultra‐low concentrations.^[170]^ These findings suggest that GO and Janus graphene nanosheets can play a crucial role in thermal EOR techniques by enhancing oil displacement, reducing interfacial tension, and increasing oil recovery rates.

#### 4.4.3 Improved Sweep Efficiency

The rGO′s graphene‐like properties make it a desirable material for various applications, including sensory and catalytic uses, which can further advance EOR technologies by boosting specific properties of composites.^[171]^ A method involving the reduction of GO to obtain rGO through UV radiation can provide a reduced form of GO with enhanced properties, potentially aiding in improved sweep efficiency during EOR processes.

#### 4.4.4 Reduction of Interfacial Tension

The combination of surfactant‐like polymers derived from GO with coupling agents like alkylamines has been proven to enhance oil recovery by reducing interfacial tension at the oil‐water interface.^[164]^ Incorporating Fe_2_O_3_‐SiO_2_ hybrid nanoparticles supported with surfactants has substantially reduced interfacial tension, further enhancing oil production rates.^[172]^ These findings collectively highlight the valuable role of graphene‐based materials in reducing interfacial tension and improving oil recovery processes.

#### 4.4.5 Environmentally Friendly Solutions

Advancements in the synthesis and functionalization of graphene derivatives, such as GO and rGO, have shown great promise in enhancing their biocompatibility and biodegradability.[^173^, ^174^] These modifications allow for tailoring the properties of graphene‐based materials to suit specific applications in biomedicine, water purification, and industrial wastewater treatment .[^174^, ^175^] By surface modifying graphene with compounds like antimicrobials, metals, and polymers, researchers have improved the biocompatibility and antimicrobial properties of these materials, making them suitable for various biomedical applications .^[174]^ The exceptional properties of graphene‐based nanomaterials, such as high specific surface area and superior adsorption capabilities, can reduce the need for additional chemicals in processes like EOR, thereby minimizing the environmental footprint and overall environmental impact.^[175]^

## 5 Challenges and Future Directions

### 5.1 Challenges

While biomass‐based CDs hold significant potential for EOR, several challenges must be addressed.

#### 5.1.1 Cost‐Effective Production

Cost‐effective and scalable methods for producing biomass‐based CDs are essential for their extensive application.^[176]^ Using green biomass resources as a carbon source offers an economically feasible synthetic route for large‐scale CD production.^[177]^ Various methods, such as hydrothermal/solvothermal, microwave‐assisted, and magnetic hyperthermia microfluidic, enable large‐scale preparation of CDs with controlled synthesis, enhancing their potential for widespread use.^[178]^ Unlike the expensive production of CNTs and graphene, biomass‐derived CDs offer a more affordable and renewable alternative for applications in sensing, bioimaging, drug delivery, and nanoelectronics.[^177^, ^179^] By exploring innovative approaches and functionalization techniques, biomass‐based CDs can address the cost and scalability challenges associated with other carbon‐based materials, paving the way for their broader adoption in various fields.

#### 5.1.2 Stability and Dispersion

Achieving stable dispersion of CNTs in carrier fluids is crucial for their effective application in various industries.^[180]^ The challenge lies in overcoming the hydrophobic nature of CNTs′ surfaces, which can lead to aggregation and hinder performance.^[180]^ Researchers have been focusing on designing novel dispersants to enhance the dispersion of CNTs, especially in solvents like water, methyl, and alcohol‐based organic solvents.^[181]^ Understanding the interplay between dispersion stability and its implications on device functionality is key to maximizing the potential of CNTs in energy storage and harvesting technologies.^[182]^ By optimizing dispersion methods and utilizing appropriate functionalization techniques, the uniform dispersion of CNTs can be achieved, ultimately improving their performance in applications such as batteries, sensors, and transistors.^[180]^

#### 5.1.3 Environmental and Health Impacts

A comprehensive evaluation of the environmental and health effects of introducing nanomaterials, including graphene, into reservoirs is crucial to ensure safe application and mitigate potential risks[^183^, ^184^] . Nanomaterials have significant applications in environmental remediation, such as oil/water separation, but their introduction into reservoirs can lead to environmental concerns and toxicity issues, especially in aquatic ecosystems. The toxicity of nanomaterials at various levels, from molecular to organismal, underscores the importance of thorough assessments to understand their impact on water habitats and aquatic life. By integrating new methodologies and a weight‐of‐evidence framework, it is possible to enhance risk assessments and make informed decisions regarding introducing nanomaterials into reservoirs, ensuring the protection of human health and the environment from adverse effects[^183^, ^185^].

#### 5.1.4 Field Testing and Scalability

Extensive field trials are crucial to validate laboratory findings and optimize the application of CNTs and graphene‐based materials for EOR.[^170^, ^186^] Techniques for scaling up production and application methods of CNTs for EOR require further development, focusing on optimizing synthesis processes, reducing costs, enhancing dispersion stability, evaluating environmental impacts, and developing scalable application methods.^[170]^ Similarly, for graphene‐based EOR techniques, future research should concentrate on developing scalable methods for production and application, considering the excellent physicochemical properties of graphene for EOR applications.^[187]^ By addressing these aspects through extensive field trials and optimization processes, the practical use of CNTs and graphene in EOR can be significantly enhanced, paving the way for their successful implementation in real‐world scenarios.

### 5.2 Future Directions

#### 5.2.1 Research and Development

Continued research and development efforts are essential to optimize the properties of CDs, CNTs, graphene, and their derivative nanomaterials for specific EOR applications. Carbon nanomaterials offer unique properties like electrical conductivity, thermal stability, and chemical resistance,^[188]^ making them promising for EOR applications in unconventional oil reservoirs.^[189]^ However, challenges such as structural nonhomogeneity and indefinite fabrication hinder their widespread implementation.^[190]^ Field data on nanomaterial use in unconventional reservoirs is limited, emphasizing the need for further research to enhance recovery rates and economic efficiency.^[189]^ A deeper understanding of nanomaterial behavior in EOR applications can be achieved by conducting physical and numerical simulation experiments optimizing their performance and applicability in the industry.^[189]^

#### 5.2.2 Field Trials

Extensive field trials are crucial to validate lab‐scale findings and showcase the efficacy of eco‐friendly carbon‐based nanomaterials like CDs, CNTs, graphene, and their derivatives on a commercial scale. Using CDs and CNTs derived from plant biomass offers a sustainable and renewable approach, with excellent adsorption capabilities for pollutants like heavy metal ions in water treatment processes. These nanomaterials′ unique physical and chemical properties make them ideal candidates for creating a pollution‐free environment and advancing green technologies. Field trials will provide valuable insights into these eco‐friendly carbon‐based materials′ scalability, efficiency, and real‐world applicability, paving the way for their commercial utilization in various industries.

#### 5.2.3 Cost Reduction

To make eco‐friendly technologies of CDs, CNTs, graphene, and their derivative nanomaterials more accessible, researchers focus on developing cost‐effective production methods using green synthesis approaches. Studies emphasize the importance of utilizing natural, renewable, and low‐cost waste resources to fabricate carbon‐based nanoparticles, ensuring sustainability and affordability.[^191^, ^192^, ^193^] These materials offer unique features such as high efficiency, selectivity, and stability, making them ideal for applications in water treatment, biomedicine, energy storage, catalysis, and sensing.[^191^, ^194^] By exploring novel synthesis techniques based on waste materials and natural precursors, researchers aim to reduce greenhouse gas emissions, harmful material extraction, and overall production costs, thus promoting the development of green technologies for a more sustainable future.[^191^, ^193^]

#### 5.2.4 Environmental Safety

To ensure environmentally safe use in EOR and disposal of eco‐friendly technologies like CDs, CNTs, graphene, and their derivative nanomaterials, it is crucial to consider their potential risks and mitigation strategies. Research has shown that carbon‐based materials, including CNTs and graphene, exhibit exceptional oil separation capabilities and reusability, making them effective in oil‐water separation processes.^[126]^ The synthesis of nanohybrids like GO‐silica nanocomposites has proven eco‐friendly and effective in altering reservoir wettability for EOR, showcasing their potential as environmentally friendly agents in the oil industry.^[7]^ These nanomaterials can be harnessed safely for EOR applications, ensuring minimal environmental impact and maximum efficiency in oil recovery processes by focusing on stability, retention, and proper disposal methods.

## Conclusions

Integrating CDs, CNTs, graphene derivatives, and their composite materials presents a novel and promising opportunity for advancing GEOR methods within the petroleum sector. Through the strategic utilization of the collective benefits offered by these various materials, there exists the potential to craft bespoke solutions that are finely tuned to address the specific complexities and obstacles encountered within individual reservoirs. The introduction of functionalized carbon nanomaterials and hybrid nanocomposites stands out as a particularly impactful advancement, capable of substantially enhancing the efficiency of oil displacement processes and serving as invaluable instruments for prolonging the productive lifespan of current reservoirs while optimizing oil retrieval rates. The ongoing pursuit of further exploration and innovation in this domain holds the promise of yielding groundbreaking, economically feasible, and ecologically sound enhanced oil recovery technologies, which could potentially bring about a transformative shift within the realm of petroleum production.

## Conflict of Interests

The authors declare no conflict of interest.

## Biographical Information

*Md Ruhul Amin Foisal received his M.Eng. degree in Petroleum Engineering from Bangladesh University of Engineering and Technology (BUET) in 2015. He is pursuing the Ph.D. degree in the Department of Chemistry at BUET under the supervision of Prof. Dr. Abu Bin Imran. His current research interest mainly focuses on the computational modeling of polymeric matrices to predict and optimize glass transition temperatures*.

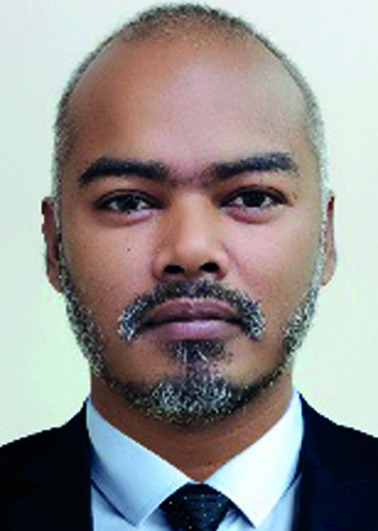

## Biographical Information

*Professor Dr. Abu Bin Imran received his Ph.D. from the Department of Molecular Design and Engineering of Nagoya University, Japan. Dr. Imran served as a Designated Assistant professor there until October 2011. He awarded a Fulbright fellowship in the Department of Chemical and Biomolecular Engineering, Rice University, USA in 2021. Currently, he is a Professor and Head of the Department of Chemistry, Bangladesh University of Engineering and Technology (BUET). Professor Imran's current research interests are to design and fabricate “smart” soft materials including polymers, colloids, liquid crystals, elastomers, nanocomposites, hydrogels and their applications in energy, biomedicine, wastewater treatment, sensors, and so on*.

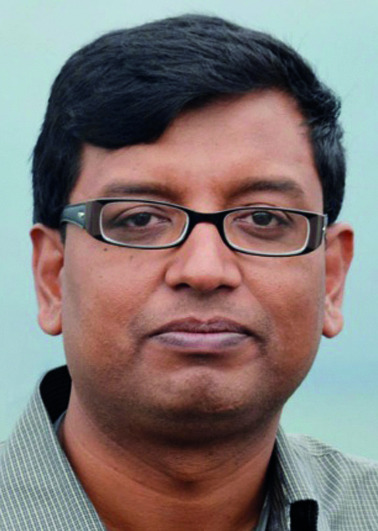

## Biographical Information

*Professor Dr. Al‐Nakib Chowdhury has received B.Sc. (Hons) and M.Sc. from the University of Dhaka, Bangladesh, Ph.D from Hiroshima University and Post‐doc from Tokyo Institute of Technology, Japan. Dr. Chowdhury is a senior Professor of Chemistry department and Dean of Science at BUET, Bangladesh. He also served as the Vice Chancellor of PUST, Bangladesh. His research interest includes energy, environment, nano scale materials, sensors, battery, solar cell, supercapacitor, CO2 capture, water splitting and catalysis. Dr. Chowdhury authored numerous high‐impact scientific papers, books, and book chapters (http://nakib.buet.ac.bd/). He is associated with a number of reputed professional societies both in Home and abroad*.

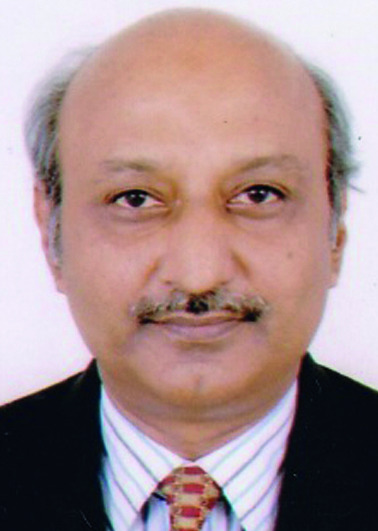

## Data Availability

The data that support the findings of this study are available from the corresponding author upon reasonable request.
